# RNA Interference-Mediated Knockdown of Male Fertility Genes in the Queensland Fruit Fly *Bactrocera tryoni* (Diptera: Tephritidae)

**DOI:** 10.3390/insects9030096

**Published:** 2018-08-10

**Authors:** Carlos Cruz, Alison Tayler, Steve Whyard

**Affiliations:** Department of Biological Sciences, University of Manitoba, Winnipeg, MB R3T 2N2, Canada; carlos.cruzlopez@umanitoba.ca (C.C.); alison.tayler@umanitoba.ca (A.T.)

**Keywords:** SIT, RNAi, *Bactrocera*, fertility, fecundity

## Abstract

The Queensland fruit fly, *Bactrocera tryoni,* is Australia’s most important horticultural pest. The Sterile Insect Technique (SIT) has been used to control this species for decades, using radiation to sterilize males before field-release. This method of sterilization can potentially reduce the insects’ abilities to compete for mates. In this study, RNA interference (RNAi) techniques were examined for their potential to sterilize male *B. tryoni* without adversely affecting mating competitiveness. *B. tryoni* adults were injected or fed double-stranded RNAs (dsRNAs) targeting spermatogenesis genes (*tssk1*, *topi* and *trxt*); quantitative reverse-transcriptase PCR analyses confirmed that transcript levels were reduced 60–80% for all three genes following injections. Feeding produced a significant gene knockdown for *tssk1* and *trxt* after three days, but interestingly, two genes (*trxt* and *topi*) produced an excess of transcripts after 10 days of feeding. Despite these fluctuations in transcript levels, all three dsRNAs impacted the fecundity of treated males, with *tssk1*- and *topi*-dsRNA-treated males producing 75% fewer viable offspring than the negative controls. Mating competition assays demonstrated that dsRNA-treated males can actively compete with untreated males. These findings suggest that RNAi technology could serve as an alternative to radiation as a means of sterilizing these insects in an SIT program.

## 1. Introduction

The Queensland fruit fly, *Bactrocera tryoni* (Diptera: Tephritidae), is the most important horticultural pest of eastern Australia, and is known to infest more than 100 native and introduced host plants [1]. The polyphagous nature of this insect, the climatic suitability of many regions within the country, and the expansion of cultivated fruit crops have all contributed to this species’ success. The Sterile Insect Technique (SIT) has been used for the management of *B. tryoni* in Australia since the 1960s [2]. The technique is considered an ideal biological control method for pest insects, since it is species-specific and does not involve the dissemination of chemical pesticides into the environment [3]. It is based on the release of large numbers of sterile males that can effectively compete with wild males for mates, thereby reducing the number of viable offspring produced [4].

SIT has been used successfully in the eradication or reduction of a diverse range of invasive and endemic pest insects, including the new world screwworm fly (*Cochliomyia hominivorax*) from continental North America to Colombia [5], the tsetse fly (*Glossina* sp.) in Zanzibar [6], and the codling moth *Cydia pomonella* in Canada [7]. SIT programs have also been effective in controlling tephritid pests of horticulture in various countries, including the Mediterranean fruit fly (*Ceratitis capitata*) in North America [8], the Mexican fruit fly *Anastrepha ludens* in Mexico [9], the melon fly *B. cucurbitae* in Japan [10], and *B. tryoni* in Australia [2].

In Australia, an extensive SIT program has enabled the establishment of the Fruit Fly Exclusion Zones, which helps to protect the country’s multibillion-dollar horticultural industry. Currently, the SIT program relies on a radiation-based sterilization method for *B. tryoni* management, releasing hundreds of thousands of sexually-immature, irradiated adult insects of both sexes within each fruit-growing district [4]. While high density mass-rearing conditions have been attributed to reduced insect fitness [11], the method of sterilization may also compromise insect performance. In various tephritids, including *B. tryoni*, sub-lethal radiation doses may also adversely affect non-reproductive tissues and could therefore reduce the treated male’s mating competitiveness, in both small lab-bred populations [12] and in mass-reared insects [13,14,15]. RNA interference (RNAi) knockdown technologies could potentially provide an alternative sterilization approach for those species that demonstrate any negative impacts from radiation treatments. RNAi, which is a sequence-specific post-transcriptional gene silencing process elicited by double-stranded RNA (dsRNA), has been used recently to sterilize or significantly reduce the fecundity of male mosquitoes [16,17,18] and fruit flies [19,20], and could provide a means of mass producing sterile males without adversely affecting other aspects of their physiology. The effectiveness of this approach will be dependent upon the development of easy and affordable methods of administering the dsRNA to a large number of insects. For many RNAi studies in insects, dsRNA has been directly injected into the insect’s hemocoel [21,22,23], but for higher throughput delivery of dsRNA, addition of dsRNA to the insect’s diet is a more practical option.

In the Oriental fruit fly *B. dorsalis*, ingested dsRNA knocked down transcript levels more than 90% [19,20], including genes associated with spermatogenesis. Spermatogenesis genes are obvious candidates to sterilize male insects when targeted with RNAi. However, not all spermatogenesis genes, when knocked down, will necessarily sterilize the male and still ensure that his mating drive and competitive behaviors remain fully intact [17]. Among the many male gametogenesis genes that would likely impact male fertility, a few have been previously considered as suitable targets for RNAi in different insects. *Testis-specific serine/threonine-protein kinase 1* (*tssk1*) encodes a protein involved in post-meiotic chromatin remodeling in insects [24], and sterility has been observed in *tssk1* mutants in mice [25]. *Matotopetli* (*topi*), encodes a transcription factor regulating sperm differentiation; mutations in this gene are known to cause sterility in *Drosophila* flies [26]. *Theoredoxin T* (*trxt*) is a testis-specific gene whose product plays a critical role in the regulation of intracellular redox homeostasis in the germ line [27]; in addition, mutants of the female variant of *trxt*, *deadhead*, caused female sterility.

Ingested dsRNA has been observed to knock down gut-specific transcripts in *B. dorsalis* larvae [28]. However, feeding spermatogenesis-specific dsRNAs to *B. dorsalis* has previously only been attempted in adults [19,20]. In *B. tryoni*, the pupation period is quite lengthy (~10 days), and effective RNAi-mediated knockdown of spermatogenesis gene transcripts would therefore require persistence of the dsRNA within the developing insects for almost three weeks. Hence, in this study, we focused our efforts on examining the efficacy of oral delivery of dsRNA targeting the three spermatogenesis genes in *B. tryoni* adults, with the aim to evaluate this technology’s potential to produce sterile males suitable for SIT control of this particular pest species. By feeding adults rather than larvae, we could avoid issues of possible dsRNA instability within the larval diet, and could administer relatively high concentrations of dsRNA in small volumes of liquid diet to the adults. Although our results did not show complete male sterility, we found that this method of dsRNA delivery resulted in a significant reduction in male fecundity. With further improvements in RNAi efficacy, a dsRNA-based method of sterilization may provide an effective alternative to radiation in *B. tryoni* SIT programs. 

## 2. Materials and Methods

### 2.1. Insect Culture

*B. tryoni,* kindly provided by Dr. Solomon Balagawi (Elizabeth Macarthur Agriculture Institute, Australia) were derived from wild flies reared from fruits collected in 2013 from Griffith, and Gosford, NSW, Australia. Adult flies were reared at 28 °C, 75% relative humidity with a photoperiod of 14:10 h (light:dark) and were provided sugar cubes and water. A Torula yeast paste was also provided to promote egg development. Eggs were laid on Macintosh apple skins and transferred to a carrot-based artificial medium [29]. Wandering larvae were transferred to Petri dishes with autoclaved sand to allow larvae to pupate, and pupae were then transferred to the colony cages (30 cm × 30 cm × 30 cm) or placed in individual cotton-stoppered vials (25 mL) for treatment.

### 2.2. RNA Isolation

Adult flies were dissected in PBS to collect gonads, male accessory glands, heads, and abdomens. Tissues were immediately transferred to 100 µL of Lysis Buffer with 2% β-mercaptoethanol and stored at −80 °C until required. Total RNA was extracted from these tissues and from different developmental stages (eggs, 2nd, 3rd, and 4th instar larvae, pupae, and adults) using QIAshredder (Qiagen, Valencia, CA, USA) columns to homogenize tissues and a GeneJET RNA purification kit (Thermo Fisher Scientific, Waltham, MA, USA). Contaminating genomic DNA was removed using an RNase-free DNase I (Thermo Fisher Scientific) treatment. RNA was quantified and purity was assessed using a Biochrom NanoVue UV-Vis spectrophotometer (Cedarlane Laboratories, Burlington, ON, Canada).

cDNA was synthesized with a qScript cDNA Supermix kit (Quanta Biosciences, Beverly, MA, USA), according to the manufacturer’s protocol. The resulting cDNAs were then PCR amplified using a Lucigen EconoTaq PLUS 2× Master Mix (following manufacturer’s protocol) and specific primers (Table S1, Supplementary Information), and subsequent 1.5% agarose gel electrophoresis. Absence of contaminating genomic DNA was confirmed using no reverse transcriptase in the negative control reactions.

### 2.3. RT-PCR Analysis

Quantitative RT-PCR was used to determine the expression patterns of genes within *B. tryoni* tissues and developmental stages, and to assess impacts of the dsRNA treatments. The qRT-PCR reactions were performed using primers (Table S1) designed from sequences acquired from the assembled *B. tryoni* genome database (http://www.ncbi.nlm.nih.gov/genome/15403). As an internal control, a fragment of the *actin-2* gene (NCBI Reference Sequence: XM_011203913) was also amplified to normalize the amount of cDNA added to the qRT-PCR reactions. QRT-PCR amplifications were performed with SsoFast Evagreen Supermix (BioRad, Mississauga, ON, Canada) according to the manufacturer’s specifications using the BioRad CFX Connect Real-Time PCR System. Melting curve analyses were performed to ensure specificity and consistency of all PCR-generated products. Controls without RT were included to confirm that genomic DNA was thoroughly removed. All reactions were repeated in duplicate (experimental replicates) and three biological replicates were performed for each gene targeted with dsRNA and to examine tissue and stage specificity. Quantification of the transcript levels of each gene in untreated insects was calculated using the 2^−ΔCT^ method [29], comparing the gene of interest’s transcripts to that of *actin*. RNAi-mediated knockdown of transcripts was calculated using the 2^−ΔΔCT^ method [30], comparing expression in specific dsRNA-fed insects to *control*-dsRNA fed insects.

### 2.4. Preparation of dsRNA

For each gene selected for RNAi targeting, PCR primers were designed to amplify fragments of ~350 bp in length, using gene-specific primers (RNAi primers in Table S1). The PCR products were ligated into the cloning vector pJET/blunt, and later excised from pJET using XbaI and XhoI restriction enzymes, then ligated into a similarly-digested plasmid pL4440, a vector possessing convergent T7 promoters. DNA templates for in vitro transcription of each of the gene fragments in pL4440 were PCR-amplified using the following pL4440-specific primers: pL4440F (ACCTGGCTTATCGAA) and pL4440R (TAAAACGACGGCCAGT). PCR products were purified using a GeneJET Gel Extraction Kit. The MEGAscript RNAi kit (Ambion, Austin, TX, USA) was then used for in vitro transcription and purification of dsRNAs, following the manufacturer’s protocol and suggested reaction conditions. DsRNA targeting a non-*B. tryoni* gene, *green fluorescent protein* (*gfp*), was used as negative control in all trials.

### 2.5. Delivering dsRNA to B. tryoni Adult Males

dsRNA solutions were diluted with molecular grade water to a concentration of 1.0 µg/µL. Ten freshly eclosed male adults (less than 12 h) were injected through the dorsal right side of the thorax with 2.0 µL of dsRNA solution using 1 mm borosilicate glass needles (pulled using a Flaming-Brown Micropipette Puller, Sutter Instruments Co., Novata, CA, USA) and then placed into individual *Drosophila* vials (25 mL). This particular dose was selected based on effective transcript knockdown observed in the closely-related species *B. dorsalis* [21,22]. To assess for RNAi, the insects were allowed to develop for three days after injection, fed on a 10% sugar water solution diet. RNA was then extracted from the 10 individual dissected pairs of testes (or entire male reproductive tract for measurements of male accessory gland (MAG) gene transcripts) and subjected to qRT-PCR analyses as described above, where the transcription levels of the genes selected were compared to control flies that were injected with *gfp*-dsRNA.

Another 10 male adults were also placed in individual vials and fed daily with a dose of 2.0 µL of dsRNA dissolved in 10 µL of 10% sugar water for three or 10 days (providing 6 or 20 μg of dsRNA to each insect over the feeding time period). The 12 µL droplet of dsRNA-sugar water solution (only food source for 24 h) was placed at the bottom of the vial daily, and most insects fed immediately, consuming most if not all of the droplet within minutes. Thereafter, RNA from dissected testes was extracted for subsequent qRT-PCR analysis. To avoid evaporation of the droplets, vials were kept in a closed plastic container lined with wet paper towels, and no evaporation was observed in control vials containing no flies.

### 2.6. B. tryoni Fertility Assays

Twenty males previously treated with dsRNA for a period of 10 days were each provided two virgin females of the same age and kept in 750 mL plastic cups for one week. Flies were provided 10% sugar water ad libitum and Torula yeast paste to promote egg development. After three days, apple skins were provided every second day over a one-week period to allow females to lay their eggs and thereby assess fertility and fecundity. Apples with eggs were transferred to the carrot-based artificial media and incubated at 28 °C for five days. Viable larvae were then counted to assess fecundity. If no eggs were produced, or if all eggs failed to hatch, the dsRNA-treated insect was considered sterile.

The 750 mL plastic cups were also used for small population mating competitions with 10 males and 10 females. In these mating competition assays, varying proportions of untreated control males to treatment males (10:0, 9:1, 1:1, 1:9 and 0:10) were mixed with 10 females. After three days, the insects were provided pieces of apple for a period of one week. The apples with eggs were transferred to the carrot-based media and incubated at 28 °C for five days, and viable larvae were then counted to assess progeny production.

### 2.7. Statistical Analysis

Significant differences between treatments and controls during the spermatogenesis genes knockdown trials (through both injections and oral delivery) were evaluated using a *t*-test for two independent sample groups. A Tukey test was performed to analyze the average of the total number of larvae per dsRNA-treated male during the mating assays, and to detect specific differences among treatment groups. Normality and Homogeneity of variances were tested using Kolmogorov-Smirnov test and Levene’s test, respectively. In cases where variables did not meet the normality and/or homogeneity premises, they were log_10_ transformed. If after variable transformation, the premises were still not met, then non-parametric methods were used. All statistical analyzes were performed in the STATISTICA 7.0 software (Dell Software, Round Rock, TX, USA) with a significance level of 0.05.

## 3. Results

### 3.1. Target Genes Identification and Tissue-Specificity

**Figure 1:**
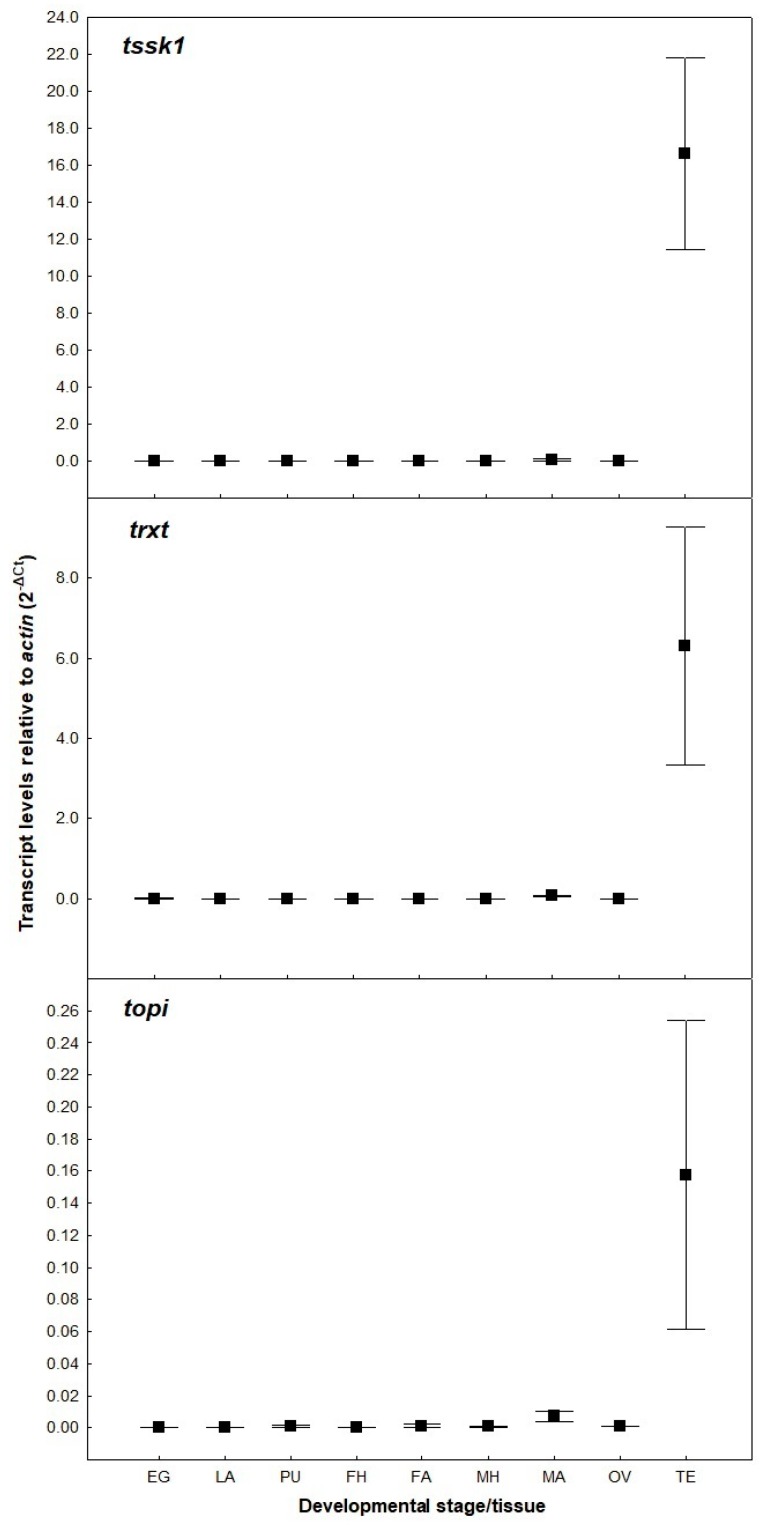
Transcript levels of putative spermatogenesis genes, relative to *actin*, in different tissues and developmental stages of *Bactrocera tryoni* (EG: eggs; LA: larvae; PU: pupae; FH: female heads; FA: female abdomens; MH: male heads; MA: male abdomens; OV: ovaries; TE: testes). Values represent the means (markers) and standard errors (whiskers) of three biological replicates.

Three putative spermatogenesis genes (*tssk1*, *topi* and *trxt*) were identified from the *B. tryoni* genome based on their high level of sequence identity to those described in *B. dorsalis* and *D. melanogaster* [24,25,26,27]. All three *B. tryoni* nucleotide sequences showed more than 95% identity with sequences of *B. dorsalis* and more than 70% with sequences of *D. melanogaster* (Table S2). To ensure that RNAi-mediated silencing of the target genes would not disrupt the development of organs other than testes, their expression patterns in different tissues of *B. tryoni* were determined using qRT-PCR. These analyses confirmed that the three genes are highly expressed only in the adult testes (Figure 1), showing no significant expression in any other tissues or developmental stages examined.

### 3.2. RNAi-Mediated Knockdown of Target Genes

**Figure 2:**
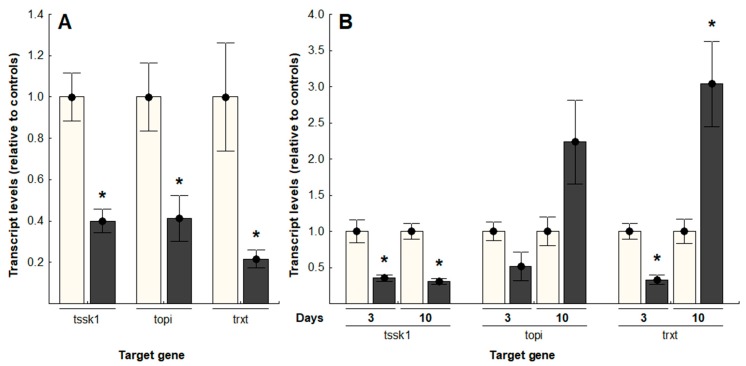
Transcript levels of *tssk1*, *topi* and *trxt*, relative to controls, in *Bactrocera tryoni* testes after dsRNA delivery: (**A**) Injected dsRNA; (**B**) Orally delivered dsRNA. Values represent the means (bars) and the 96% standard errors (whiskers) of 10 biological replicates; asterisks indicate significant differences, *t*-test, *p* < 0.05 (White bars: Negative controls, grey bars: gene-specific dsRNA treatments).

Hemocoel injections of dsRNA targeting *tssk1*, *topi* and *trxt* in *B. tryoni* produced significant transcript knockdown of all three genes in adults, three days post-injection (Figure 2A); *tssk1* showed a 60.1% knockdown (*t* = 4.44, *p* < 0.05); *topi*, 58.8% knockdown (*t* = 2.82, *p* < 0.05); and *trxt* had the greatest reduction of transcript levels, with 78.3% knockdown (*t* = 3.80, *p* < 0.05), relative to the negative controls treated with *gfp*-specific dsRNA. Oral delivery of dsRNA, accomplished by daily doses of 2.0 µg dsRNA in sugar water, achieved a significant reduction of transcript levels for *tssk1* and *trxt* after three days of feeding, while *topi* transcripts were not significantly affected (Figure 2B). After 10 days of dsRNA feeding, only *tssk1* transcripts remained significantly reduced (69.1% reduction; *t* = 5.77, *p* < 0.05), relative to controls (*t*-test, *p* < 0.05). In contrast, flies fed dsRNA targeting either *topi* or *trxt* showed approximately two- to three-fold increases in transcript levels, compared to control flies (*topi*-treated flies, *t* = −1.91, *p* = 0.07; *trxt*-treated flies, *t* = −3.75, *p* < 0.05).

### 3.3. Effect of Gene Silencing on Male Fecundity

**Figure 3:**
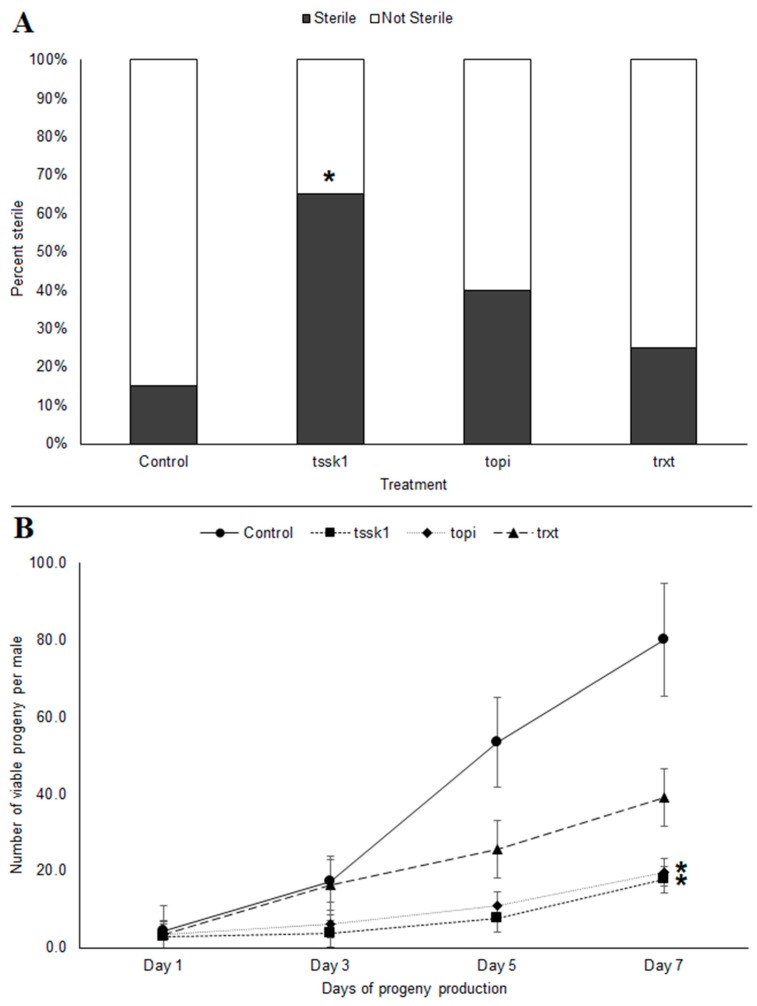
Reproductive fitness of *tssk1*-, *topi*- and *trxt*-dsRNA treated males of *Bactrocera tryoni* over a 7-day period post-treatment: (**A**) Percentage of males exhibiting complete sterility; (**B**) Fecundity of dsRNA-treated males, based on number of viable progeny produced over a one-week period. Values represent the means (markers) and the 96% standard errors (whiskers) of 20 biological replicates; asterisks indicate significant differences (*p* < 0.05).

Feeding *B. tryoni* males *tssk1*-dsRNA for 10 consecutive days resulted in an increased frequency of sterile males (χ^2^ = 8.45, *p* < 0.05) (Figure 3A). Interestingly, even though *trxt*-dsRNA showed no knockdown of transcripts at day 10, these males also exhibited reduced fecundity, which suggests that the earlier knockdown (observed at day 3) perturbed gene expression sufficiently to impact their overall reproductive fitness. The cumulative production of progeny over a week-long period following the 10 days of previous dsRNA feeding resulted in an overall reduction in viable progeny for all three dsRNAs tested. Males fed *tssk1*- and *topi*-dsRNAs showed almost similar reduction in viable progeny, with 77.8% and 75.4% reductions in offspring at day 7, respectively (K-W test, *p* < 0.05), relative to the negative controls. In *trxt*-dsRNA fed males, progeny production was still reduced 51.1% by day 7 (K-W test, *p* = 0.27) (Figure 3B).

### 3.4. Effect of Gene Silencing on Male Competitiveness

**Figure 4:**
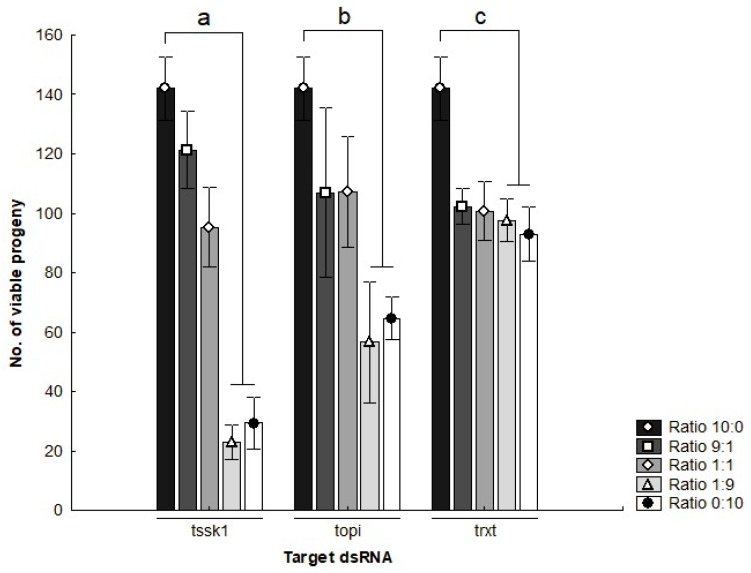
Number of viable progeny of *Bactrocera tryoni* produced in cages seeded with different ratios of dsRNA-treated males (Negative controls: gene-specific dsRNA treatments). Values represent the means (bars) and the 96% standard errors (whiskers) of three biological replicates; letters denote statistically significant differential responses relative to the control treatments, *t*-test (*p* < 0.05).

The mating competitiveness of dsRNA-treated males was assessed by mixing different proportions of dsRNA-treated males into small populations with untreated males and females. Males treated with *tssk1*- or *topi*-dsRNA significantly reduced progeny production by approximately 85% and 60% respectively, relative to control populations, when seeded at densities of 1:9 and 0:10 (*gfp*:target gene) (*t*-test, *p* < 0.05) (Figure 4). Cages seeded with high ratios of *trxt*-dsRNA-treated males showed the lowest reduction in viable offspring with only 30%, compared to control cages (*t*-test, *p* < 0.05).

As MAG proteins can also impact mating success in *B. tryoni* [31], transcript levels of two MAG genes (*protein disulfide isomerase* (*diso*) and *odorant binding protein 2* (*obp2*) (Table S3)) were analyzed using qRT-PCR after the 10 day dsRNA treatment. Interestingly, the dsRNA treatments caused significant overexpression of these two MAG protein-encoding genes in most males (Figure S1A, B), relative to the negative control treated males.

## 4. Discussion

In this study, we found that feeding dsRNAs to adult male *B. tryoni* can induce RNAi-mediated knockdown of testis-specific genes. Variation in RNAi efficacy was observed for the different genes targeted, but nevertheless, the dsRNAs were effective at reducing male fertility and fecundity. While complete sterility was not achieved in all males for any of the dsRNA treatments examined here, all three dsRNAs were effective in causing significant reductions of overall progeny production in mating competition assays. These findings suggest that with some further improvements, oral delivery of dsRNA may provide an alternative to radiation-induced sterility in *B. tryoni* and other tephritid SIT programs.

Our experimental results demonstrate for the first time that RNAi is both operational and systemic in *B. tryoni*, as the ingested dsRNA traversed from the gut to the gonad to knock down testis-specific transcripts. This is not entirely surprising, as systemic RNAi has been observed in other tephritids [19,20]. At least two of the three dsRNAs (targeting *tssk1* and *trxt*) induced a significant knockdown of the target transcripts within the testis after three consecutive days of dsRNA feeding. By day 10, however, two genes (*topi* and *trxt*) showed no evidence of knockdown, but instead, they exhibited over-expression, relative to the negative controls. This observation suggests that the cells that had initially experienced RNAi-mediated knockdown of transcripts subsequently increased gene expression to compensate for the earlier reduction of both *trxt* and *topi*. The fact that the *tssk1* transcript levels did not respond similarly, but remained suppressed over the dsRNA feeding period, suggests that genes can respond very differently to dsRNA-mediated knockdown, and that variability of RNAi efficacy will be dependent on different regulatory mechanisms of each targeted gene.

Another research group has observed a similar up-regulation of *topi* following continuous dsRNA feeding in another tephritid species, *B. dorsalis* [20]. They suggested that this lack of RNAi efficiency might arise if the initial dose of dsRNA is too high, and that this refractoriness to the dsRNA may not be a sequence-specific phenomenon. In our experiments, however, we observed no evidence of RNAi refractoriness, at least when targeting *tssk1* using similar doses of dsRNA as those used in the *B. dorsalis* studies. This suggests that if refractoriness is indeed a response to continuous or large doses of dsRNA, its potency may vary, depending on the target gene. The mechanism(s) by which refractoriness might occur have not been defined, but may be dependent on the transcription rate of the target gene. In our study, we found that only the highest expressed gene, *tssk1*, was effectively and continuously knocked down by the administered dsRNA, whereas the two genes with the less abundant transcripts recovered from any initial knockdown. Perhaps the RNAi machinery can be inhibited if RISC and its associated siRNAs fail to find a sufficient quantity of target transcripts within a limited time period, but if target transcripts are plentiful, RNAi mechanisms are sustained. A broader sampling of gene targets with a range of transcription rates will be worth exploring to assess whether continuous transcript abundance does indeed play a role in modulating the stability and function of RNAi-associated activity and possible refractory behavior.

Male *B. tryoni* typically take over one week to reach sexual maturity [32], which provided us with sufficient time to deliver enough dsRNAs to impact the maturing male’s fertility. In most SIT programs, sterile males are released while the males are still young (~3 days old) [4]. Retaining the adults in a rearing facility for a somewhat longer dsRNA feeding period has both advantages and disadvantages. On one hand, retention of the insects within a factory will protect them from predation until they are needed in the fields, while on the other hand, earlier release minimizes rearing costs and allows the insects to disperse more widely before they are ready to mate. For species that mature more rapidly than *B. tryoni*, feeding young males sterility-inducing dsRNAs would not likely be very effective, as many of the sperm could have developed fully before the delivered dsRNA would have an impact. In those species, dsRNA should be more effective if fed to developing larvae, before the targeted genes are expressed. Feeding dsRNAs to *B. tryoni* larvae was not attempted here, but this type of dsRNA delivery is worth considering for future studies to assess whether the dsRNA can remain stable within a larval diet, and whether sufficient dsRNA can persist through the lengthy pupation period for this species, which was approximately 10 days. Evidence of dsRNA persistence through insect development has not yet been fully explored, but has been demonstrated in a small number of holometabolous insects, such as honeybees [33], beetles [34], and mosquitoes [17]. It is anticipated that some improvements to the durability of the dsRNA, either in its chemical composition or in stabilizing additives (e.g., nanoparticles and other microcarriers) [35,36], would be required if such a method was to be fully effective. In an adult feeding regimen, it is possible to administer the dsRNA to the insects in small volumes of liquid (e.g., sucrose) diet, but to mass-feed larvae, considerably greater quantities of dsRNA may be required to attain the same phenotypic effect in the adults.

Despite observing incomplete reduction of the targeted transcripts, the dsRNAs tested in this study still affected the overall fecundity of the male insects. However, other target genes may affect the fertility more strongly than those examined here. The two dsRNAs targeting the early spermatogenesis genes, *topi* and *trxt*, failed to show sustained transcript knockdown, and their impact on male fecundity was not as potent as the dsRNAs that targeted the *tssk1* gene, which mediates chromatin condensation during late spermatogenesis. It will be interesting to determine whether other late-stage spermatogenesis genes are more likely to (1) show more sustained RNAi-mediated transcript knockdown; and (2) show more pronounced impacts on male fertility. For sterile males to be effective in an SIT program, they must still demonstrate competitive mating behavior and be able to transfer seminal fluids to the female. These fluids have been found in many insects, including *B. tryoni* [31], to reduce a female’s receptivity to subsequent matings. In our dsRNA-treated males, only two male accessory gland protein transcripts were examined, and both showed, unexpectedly, increases in accumulation, which suggests that these two proteins at least were not likely depleted in these males. Further analyses of the male accessory gland proteins produced by our dsRNA-treated males would be informative in determining their full competitiveness relative to fertile males in the field.

Currently, *B. tryoni* SIT programs rely on radiation-sterilized male insects, and are considered an effective, pesticide-free method of control. While mass-rearing conditions have been attributed to reduced male fitness [11], numerous studies also implicate radiation as a potential cause for reduced male mating competitiveness [12,13,14,15]. For a dsRNA-mediated sterilization method to be adopted, the RNAi-treated males should be more effective competitors than radiation-sterilized males. In our lab-based trials, we observed that a 9:1 ratio of dsRNA-treated males to untreated males resulted in a 5-fold reduction in the next generation. With this level of efficacy, one could only achieve population suppression, rather than complete eradication. Higher over-flooding ratios (OFRs; sterile:wild males) have been used to control other tephritids using SIT technologies [37], and for *B. tryoni* SIT programs, a report by Horticulture Australia Ltd. recommended a OFR of 100:1 to achieve effective insect control [38]. It will be worthwhile assessing a wider range of OFRs using dsRNA-treated males to determine whether RNAi technology can match the efficacy of radiation-based sterilization.

Aside from increasing OFRs, other enhancements of the RNAi technique, including (1) identifying more potent target genes; (2) improvements in stabilizing dsRNAs in dietary formulations; and (3) reducing dsRNA production costs could enable a shift from radiation sterilization to dsRNA-mediated sterilization methods. Of these three, advancements in mass production of dsRNA have already been realized, as a number of agriculture industries and dsRNA synthesis companies have developed large-scale dsRNA production systems, for a range of applications, including the production of insecticidal dsRNAs (reviewed in [39,40]). Another factor that could make dsRNA-mediated sterilization more appealing is if it could be simultaneously coupled with a sex-sorting method, to prevent females from being reared in SIT sterile male production facilities. RNAi could, for example, also be used to target the female variant of genes involved in the sex determination pathway, such as *doublesex* or *transformer*, to produce a male-biased population for field release [41]. Ideally, the elimination of females from the lab- or factory-bred populations should occur earlier in development, to reduce the costs of rearing both sexes. Feeding dsRNA to larvae has been effective in *B. dorsalis* [28] and hence, may prove effective in *B. tryoni* to prevent female development. One other issue that will need to be considered is whether dsRNA-sterilized males may regain full fertility over time. However, given that they had significantly reduced fecundity in the first 10 days of their reproductive period, this method of sterilization could still result in strong impacts on the pest populations.

## 5. Conclusions

In summary, this study’s findings demonstrate that ingested dsRNA can be used to reduce male *B. tryoni* fecundity without impairing their ability to mate. With further refinements in target gene selection, dsRNA delivery and RNAi efficacy, and more thorough exploration of population responses to dsRNA-sterilized insects, RNAi technology may provide an alternative to radiation based sterilization methods in SIT programs for this species and potentially other insect pests amenable to SIT-based control.

## Supplementary Materials

The following are available online at http://www.mdpi.com/2075-4450/9/3/96/s1, Table S1: Primers used for RT-PCR and qRT-PCR analyses, Table S2: Percentages of nucleotide similarity between the genome of *Bactrocera tryoni* and the genes of interest in *B. dorsalis* and *Drosophila melanogaster*. Sequence identity analyses were performed using the BLAST program of the National Center for Biotechnology Information (NCBI), Table S3: Percentages of nucleotide similarity between the male accessory gland genes in *Bactrocera dorsalis* and the genome of *B. tryoni*. Sequence identity analyses were performed using the BLAST program of the National Center for Biotechnology Information (NCBI), Figure S1: Transcript levels of two male accessory gland protein encoding genes, relative to actin, in *Bactrocera tryoni* after ten days of continuous oral delivery of dsRNA: (A) *Protein disulfide isomerase* (*diso*); (B) *Odorant binding protein 2* (*obp2*). Values represent the means (bars) and the 96% standard errors (whiskers) of ten biological replicates; letters indicate significant differences, Tukey test.
