# Supplementary material for: RNA Interference-Mediated Knockdown of Male Fertility Genes in the Queensland Fruit Fly Bactrocera tryoni (Diptera: Tephritidae)
Source: Insects. 2018 Aug 10;9(3):96. doi: 10.3390/insects9030096 (PMC6163526; doi:10.3390/insects9030096)
Supplement: Supplementary file 1 [file insects-09-00096-s001.pdf]

insects

Article

RNA Interference-Mediated Knockdown of Male Fertility Genes in the Queensland Fruit Fly *Bactrocera tryoni* (Diptera: Tephritidae)

Carlos Cruz, Alison Tayler and Steve Whyard *

Department of Biological Sciences, University of Manitoba, Winnipeg, MB R3T 2N2, Canada; carlos.cruzlopez@umanitoba.ca (C.C.); alison.tayler@umanitoba.ca (A.T.)
* Correspondence: Steve.Whyard@umanitoba.ca; Tel.: +1-204-474-9418

Supplementary Materials

1. Tables

Table S1. Primers used for RT-PCR and qRT-PCR analyses.

Gene	Primer	Primer Sequence
**Putative testis-specific genes**		
Testis-specific serine/threonine kinase 1 (tssk1)	QFFtssk RNAi F	TTTCGACAACCTCCAATCGCCAAC
	QFFtssk RNAi R	CGCATAGGAACCCTCGCCAATTT
	QFFtssk qRT F	CGCCGCCTCCTCAACTAAATGAT
	QFFtssk qRT R	CCTTGGTCCACGACCAGACAAAT
Matotopetli (topi)	QFFtopi RNAi F	ACGCTTCTTAACTGGCTCCGTTT
	QFFtopi RNAi R	GGCATTATTGGCATGCTGCTGTT
	QFFtopi qRT F	CATTTGGATGCGAACGCTCGTTTAATG
	QFFtopi qRT R	GGCATTATTGGCATGCTGCTGTT
Thioredoxin T (trxt)	QFFtrxt RNAi F	GATCTGGATAAGAAGCTGGAGGAAGC
	QFFtrxt RNAi R	TCGGCTTCCACAATTTCTGGCATATT
	QFFtrxt qRT F	GCCGAGAAAGCGATTGTGTTGAAAG
	QFFtrxt qRT R	TGAGTTGCCGCCAACGAATACA
**House-keeping reference gene**		
Actin	QFFact qRT F	CCATGCCATTCTCCGTTTGGATTTG
	QFFact qRT R	AGCTGTGGTGGTGAACGAGTAG
**Accessory gland protein genes**		
Protein disulfide isomerase (diso)	QFFdiso qRT F	AGGGCGAACACACTGTTGAGAA
	QFFdiso qRT R	GATCTTGGAAGCTGATTCGTGGTTGA
Odorant binding protein 2 (obp2)	QFFobp2 qRT F	ATCATGCCGGTCACTCCGATTATG
	QFFobp2 qRT R	TCTGGAAAGCTCCACTGCTTGT

Table S2. Percentages of nucleotide similarity between the genome of *Bactrocera tryoni* and the genes of interest in *B. dorsalis* and *Drosophila melanogaster*. Sequence identity analyses were performed using the BLAST program of the National Center for Biotechnology Information (NCBI) (http://blast.ncbi.nlm.nih.gov/Blast.cgi).

Gene	% of nucleotide identity with *B. dorsalis*	% of nucleotide identity with *D. melanogaster*
Testis-specific serine/threonine kinase 1 (tssk1)	97.5 (XM_011212727.2)	75.0
Matotopetli (topi)	97.1 (XM_011212200.2)	78.0
Thioredoxin T (trxt)	96.4 (XM_011200823.2)	70.0

Table S3. Percentages of nucleotide similarity between the male accessory gland genes in *Bactrocera dorsalis* and the genome of *B. tryoni*. Sequence identity analyses were performed using the BLAST program of the National Center for Biotechnology Information (NCBI) (http://blast.ncbi.nlm.nih.gov/Blast.cgi).

**Gene**	**% of nucleotide identity with *B. dorsalis***
Protein disulfide isomerase (diso)	97.7
Odorant binding protein 2 (obp2)	84.5

2. Figures

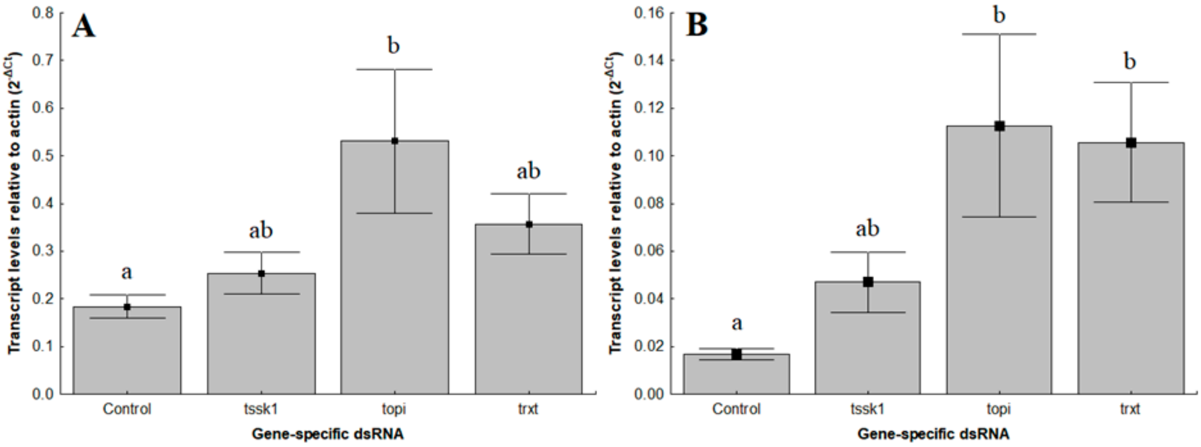

Figure S1. Transcript levels of two male accessory gland protein encoding genes, relative to actin, in *Bactrocera tryoni* after ten days of continuous oral delivery of dsRNA: (A) *Protein disulfide isomerase* (*diso*); (B) *Odorant binding protein 2* (*obp2*). Values represent the means (bars) and the 96% standard errors (whiskers) of ten biological replicates; letters indicate significant differences, Tukey test.

© 2018 by the authors. Submitted for possible open access publication under the terms and conditions of the Creative Commons Attribution (CC BY) license (http://creativecommons.org/licenses/by/4.0/).
